# Protective efficacy of dapagliflozin against schistosomiasis mansoni-induced liver pathology: an in vivo study

**DOI:** 10.1038/s41598-026-55860-8

**Published:** 2026-06-06

**Authors:** Mona K. Hegazy, Rokia Masoud, Basma H. Othman, Samar M. Alhusseiny

**Affiliations:** 1 Department of Medical Parasitology, Faculty of Medicine, Mansoura University, 2 El-Gomhouria Street, Mansoura, 35516 Egypt; 2 Department of Anatomic Pathology, Faculty of Medicine, Mansoura University, Mansoura, 35516 Egypt; 3 Medical Experimental Research Center, Faculty of Medicine, Mansoura University, Mansoura, 35516 Egypt

**Keywords:** *Schistosoma mansoni*, Dapagliflozin, Praziquantel, Oxidative stress, Hepatic granuloma, Cleaved-caspase 3, Diseases, Drug discovery, Gastroenterology, Medical research

## Abstract

Schistosomiasis is a neglected tropical disease associated with significant morbidity and mortality. Treatment with praziquantel; the sole current medication for human schistosomiasis, is associated with incomplete resolution of *Schistosoma mansoni*-induced liver pathology. Therefore, there is a crucial need to introduce adjuvant agents that can mitigate the pathological events. This study aims to assess the hepatoprotective effects of the oral anti-diabetic drug dapagliflozin, alone and in combination with praziquantel. In the present research, combined dapagliflozin and praziquantel therapy caused significant decrease in adult worm burden and hepatic egg load, significant increase in the percent of mature and dead eggs and significant reduction in the percent of immature eggs in the small intestine, significant increase in hepatic levels of reduced glutathione and nitric oxide, significant attenuation of histopathological changes, and significant reduction in granuloma count and diameter. Additionally, immunohistochemical study of cleaved-caspase 3 revealed increased expression, compared to the untreated group. Our findings provide insight into dapagliflozin use as an advantageous adjuvant to praziquantel in alleviating schistosomal liver pathology. Future studies are needed to explore other pathways responsible for the hepatoprotective effects of dapagliflozin against *Schistosoma-mansoni*-induced liver fibrosis.

## Introduction

Schistosomiasis is a parasitic disease that causes major health and economic impacts. Although schistosomiasis burden has declined (based on historical data from 1990 to 2021, and projections for 2022 to 2030), an increase in the disease burden has been demonstrated in areas with high socio-economic levels. This insinuates a significant contribution of different factors, such as imported cases and climate change^1^.

Liver fibrosis complicates chronic liver injury and inflammation evoked by different causes, such as *Schistosoma* infection^2^. Ova trapped within liver sinusoids trigger granulomatous inflammation, fibrosis, and portal hypertension. The factors implicated in *Schistosoma*-induced liver fibrosis include the immune response (provoked by antigens released by ova), fibrogenesis, and tissue damage in response to proteases produced by adult worms and ova^3^.

Activation of hepatic stellate cells (HSCs) is the principal mechanism stimulating the synthesis of fibrous collagens and extra-cellular matrix (ECM) proteins. During fibrogenesis and tissue repair, these cells produce large amounts of matrix components and pro-inflammatory cytokines, and undergo trans-differentiation into myofibroblasts (MFs)^4^.

Inflammation is associated with increased production of reactive oxygen and nitrogen species; RONs^5^. When the natural anti-oxidant defenses are subdued by the high concentration of RONs, a state of redox imbalance, oxidative stress, permanent changes in cellular compounds, and perturbation of normal cellular signalling pathways ensues^6,7^. Oxidative stress is a primary mechanism implicated in liver fibrosis^8^; acting *via* activating HSCs, and stimulating ECM proteins and collagen accumulation by MFs^9^.

Following infection, the innate immune response serves as the first line of defense, and coordinates inflammation and cell death^10^. Apoptosis is a non-lytic, non-inflammatory cell death that is mainly controlled by caspases; the core regulators and executives of apoptosis in multicellular organisms^11,12^. Caspases are members of the cysteinyl protease family, and are also known as cysteine-dependent aspartate-specific proteases. Constitutional expression of caspases takes place during homeostatic mechanisms, in immune and non-immune cells. The executioner caspase-3 is cleaved and activated *via* the initiator caspase-8 or -9; external and intrinsic activation pathways, respectively^13^. Owing to their central role in cell death pathways, caspases have emerged as potential therapeutic candidates for a wide variety of ailments, such as inflammation, infectious diseases, and cancer^14^.

Dapagliflozin (DAPA) is an FDA-approved sodium-glucose co-transporter−2 (SGLT2) inhibitor. It belongs to the newer anti-diabetic medications used to manage type 2 diabetes mellitus^15^. The SGLT2 inhibitors act by competitively binding to glucose-binding sites on SGLT2 proteins in the renal tubules. This binding decreases tubular glucose reabsorption, and increases glucose, water, and sodium excretion in urine, with subsequent reductions in blood glucose levels and volume load^16–18^.

The hypoglycemic effects of SGLT2 inhibitors drive decreased glucose toxicity, enhanced glucose metabolism, and improved insulin sensitivity. The non-hypoglycemic protective effects of SGLT2 inhibitors include anti-inflammatory, anti-oxidant, anti-aging, and anti-fibrotic benefits, in addition to enhancing mitochondrial function^19^.

Administration of DAPA was demonstrated to alleviate liver injury induced by common bile duct ligation in rats^20^. Also, DAPA was recorded to slow down the progression of liver fibrosis in mice^21^. Besides, manifestations associated with non-alcoholic fatty liver disease in rats improved in response to DAPA therapy^22^.

Because of the previously documented hepatoprotective effects of DAPA, the present study was designed to evaluate its efficacy against hepatic schistosomiasis mansoni, employing general, parasitological, biochemical, histopathological, and immunohistochemical studies.

## Materials and methods

### Drugs and dosing protocols

Praziquantel (PZQ; Biltricide 600 mg tablets, Alexandria Co. for Pharmaceuticals & Chemical Industries, Alexandria, Egypt), and dapagliflozin; DAPA (Dapaveldactin 5 mg tablets, Liptis Pharmaceuticals, Giza, Egypt) were used in the current experimental work.

The murine dose of DAPA was calculated^23^ after the corresponding rat dose that improved biochemical parameters, and ameliorated histopathological changes in a liver fibrosis model of common bile duct ligation^20^.

Dapagliflozin was administered as 20 mg/kg, every other day (EOD) from the 4th to the 10th week post infection (WPI). Praziquantel was given 6 WPI as 500 mg/kg/day for two successive days^24^.

Dimethyl sulfoxide (DMSO) 1% and Cremophor El 2% in aqueous suspensions were used as solvents for DAPA and PZQ, respectively. Drugs were administered *via* oral gavage, using a mouse-feeding needle.

### Parasites and snail host

Infection was induced through subcutaneous injection of each mouse by 60 ± 10 cercariae of an Egyptian strain of *Schistosoma mansoni*^25^, freshly shed from *Biomphalaria alexandrina* snails. Snails were obtained from the Schistosome Biological Supply center (SBSC), Theodor Bilharz Research Institute (TBRI), Giza, Egypt.

### Study design

The sample size was calculated according to the resource equation approach, based on the acceptable range of the degrees of freedom; DF^26^.

A total of 30 laboratory-bred female BALB/c mice (aged 6–8 weeks, and weighing 20–25 gm) were bought from the SBSC, TBRI, Giza, Egypt. Mice were randomly assigned into five groups (each containing six mice at the beginning of the study):

- Group I: infected, non-treated.
- Group II: infected and treated with DMSO (as a vehicle control), EOD from the 4^th^ to the 10^th^ WPI.
- Group III: infected and treated with PZQ.
- Group IV: infected and treated with DAPA.
- Group V: infected and treated with DAPA, besides PZQ.

All animal procedures were approved by the Mansoura University Animal Care and Use Committee (MU-ACUC); code number: MU-ACUC (MED.R.25.01.54), and all methods were carried out in accordance with the relevant guidelines and regulations. All animal procedures followed the ethical principles, guidelines, and regulations of the MU-ACUC, in compliance with the ARRIVE guidelines.

Mice were housed at the Medical Experimental Research Center (MERC, Faculty of Medicine, Mansoura University) under controlled environmental conditions, with access to food and water *ad libitum.* Mice were euthanized ten WPI, using overdose of the anesthetic agent thiopental sodium (EIPICO; Sharqia, Egypt) as intra-peritoneal injection, at 120 mg/kg.

### Calculation of the mortality rate

The mortality rate was calculated using the formula: mortality rate= (number of dead mice/total number of mice per group) × 100.

### Assessment of liver and spleen indices

Livers and spleens were removed and weighed to calculate liver and spleen indices using the formula: (Liver or spleen index= liver or spleen weight×100/body weight).

### Parasitological parameters

#### Adult worm burden

Adult worms were recovered from hepatic and porto-mesentric vessel perfusates using a veterinary scalp and citrate saline (1.5% sodium citrate, 0.85% sodium chloride), for subsequent counting^25^.

#### Hepatic egg load

Potassium hydroxide (4%) was added to weighed liver fragments, and preparations were incubated overnight at 37 °C. After vortexing, a volume of 50 µ was aspirated from the homogenate, and examined microscopically for eggs. Counts were recorded three times per specimen, then the means were calculated^27^.

#### Oogram pattern

Oogram pattern was done to report different egg developmental stages (immature, mature and dead) in fragments from the terminal ileum. After rinsing, fragments (each about 1 cm) were opened lengthwise with a pair of scissors. Each fragment was slightly dried using filter paper, and placed between two slides and compressed. One hundred eggs were counted in each fragment, and from each mouse three fragments were examined, to obtain a total of 300 eggs^28^.

### Biochemical parameters

#### Assay of reduced glutathione and nitric oxide levels in liver homogenates

According to the manufacturer’s instructions, the levels of reduced glutathione (GSH) and nitric oxide (NO) were determined in liver tissue homogenates, using commercially available kits (Biodiagnostics, Giza, Egypt), CAT. No. GR 25 11, and NO 25 33, respectively.

### Histopathological study

#### Parenchymatous changes and portal tract inflammation and expansion

Liver portions close to the porta hepatis from euthanized mice were fixed in 10% neutral buffered formalin, and processed to paraffin blocks. Sections were cut (5 μm thick), and then stained with Haematoxylin and Eosin to evaluate histopathological changes.

Sections were blindly examined, and scored for:

- Lobular inflammation (according to the number of inflammatory foci): no inflammatory foci (none); 1–2/×200 (mild); up to 4/×200 (moderate); > 4/×200(marked)^29^,
- Focal necrosis and hydropic degeneration of hepatocytes: 0% (none); 1–10% (minimal); 11–30% (mild); 31–60% (moderate); > 60% affected liver cells (marked)^30^,
- Portal tract inflammation and expansion: no portal inflammation (none); scattered inflammatory cells affecting ≤ 1/3 of portal tracts (mild); increased inflammatory cells affecting1/3−2/3 of portal tracts (moderate); intense packing of inflammatory cells affecting ≥ 2/3 of portal tracts (marked)^31^,
- Inflammatory cellular infiltrate was categorized using a semiquantitative histologic evaluation (×40 magnification) in five microscopic fields of highest inflammatory density: ≤25% inflammatory infiltrate (minimal); 26–50% (mild); 51–75% (moderate); >75% (marked)^32^.

#### Hepatic granulomas

Liver sections were stained with Masson’s trichrome stain to visualize collagen fibers deposition around granulomas. Granuloma count and diameter were recorded in serial hepatic sections of ˃250 μm apart (three successive microscopic fields). An ocular micrometer was used to measure granuloma diameter. Only lobular non-confluent granulomas, surrounding single ovum in their centers were measured^33^.

### Immunohistochemical study of cleaved-caspase−3

For immunohistochemical staining, tissue sections with a thickness of 5 μm were cut on positively charged slides. The tissue sections underwent dewaxing followed by rehydration (using descending grades of alcohol). To reduce the activity of endogenous peroxidase, the sections were exposed to hydrogen peroxide/methanol 10% for 10 min. Then, they were exposed to microwave irradiation using 0.01 M sodium citrate buffer (pH 6.0) for 10 min, allowed to cool to room temperature, and then washed three times in phosphate-buffered saline (PBS), each lasting 5 min. Afterwards, the sections were autoclaved in citrate buffer for 11 min, to facilitate antigen retrieval. Subsequently, the sections were incubated with the primary antibody (Anti-cleaved-caspase−3 polyclonal antibody, GB 11532, ServiceBio, Wuhan, Hubei, China; diluted 1:600using PBS), and left overnight at a temperature of 4 °C. Later, a goat polyclonal secondary antibody was incubated with the sections for 30 min at room temperature with diaminobenzidine. In the end, the tissue sections were counterstained with Hematoxylin, dehydrated in alcohol, cleared in xylene, and mounted^34^.

The sections were examined to determine the sites of antibody positivity, and to assess immunostaining. The immunoreaction was considered positive when brownish nuclear staining was visualized. A semiquantitative system was used to evaluate the expression of cleaved-caspase-3 by calculating the percentage of positive cell expression as follows: trace: ˂ 5% positive cells; weak (1+): 5–25% positive cells; moderate (2+): 26–75% positive cells; and strong (3+): ˃ 75% positive cells^35^.

### Statistical analysis

Data were analyzed using statistical package for social sciences (SPSS) software for Windows (SPSS Inc., Chicago, USA), version 26.0. Data normality was initially analyzed using Shapiro-Wilk test. Continuous variables were presented as mean ± standard deviation for the normally-distributed data, or as median for the non-normally-distributed data. The studied groups were compared by analysis of variance (ANOVA) followed by post hoc testing using Fisher’s least significant difference (parametric data), or Kruskal Wallis (KW) test followed by Mann-Whitney test (non-parametric data).

Qualitative data were presented using number and percent, and associations were tested using Monte Carlo test when expected cell count was less than 5. The results were considered significant when *P* values were ˂0.05. Comparison was made between the infected non-treated group and treatment groups to detect any significant differences in measured variables, and among different treatment groups to evaluate the effects of using DAPA and PZQ either as mono- or combined therapy.

The percentage of reduction or increase was determined using the formula: Percentage of reduction or increase= (value of the untreated group-value of the treated group) ×100/value of the untreated group.

## Results

### Mortality rate

One mouse died in DMSO-treated group (16.67%), and in DAPA-treated group (16.67%), while no mortality was recorded in the other groups (0%).

### General parameters

#### Liver and spleen indices

Mice-administered PZQ or DAPA monotherapy showed significant reduction in liver index (by 17.59% and 14.18%, respectively), and in spleen index (by 43.75% and 27.78%, respectively), compared to infected untreated mice. Additionally, combined DAPA and PZQ therapy significantly decreased liver and spleen indices (by 16.88% and 42.36%, respectively), compared to infected non-treated group (Table 1).

**Table 1:** Liver and spleen indices among different *Schistosoma mansoni*-infected mice groups, treated with praziquantel and dapagliflozin as mono- or combined therapy.

Mice groups	Liver index ^a^	Spleen index ^a^
Infected non-treated (n = 6)	7.05 ± 0.79	1.44 ± 0.19
DMSO (n= 5)	6.89 ± 0.29	1.34 ± 0.23
PZQ (n = 6)	5.81 ± 0.97(17.59) ^*^	0.81 ± 0.35(43.75) ^*^
DAPA (n = 5)	6.05 ± 0.23 (14.18) ^*^	1.04 ± 0.05 (27.78) ^*^
DAPA + PZQ (n = 6)	5.86 ± 0.13 (16.88) ^*^	0.83 ± 0.10 (42.36) ^*^
*P*-value	*P* = 0.003	*P* ≤ 0.001

^a^ Analysis of variance (ANOVA) test. Data are expressed as means ± standard deviation

Values enclosed between parentheses represent the percentage of reduction, compared to infected non-treated group

DAPA: dapagliflozin; DMSO: dimethyl sulfoxide; PZQ: praziquantel

^*^ Significant difference against infected non-treated, and DMSO-treated groups at *P*˂ 0.05

### Parasitological parameters

#### Adult worm burden

Administration of PZQ either alone or combined with DAPA significantly reduced the females (by 80% and 93.33%, respectively), and total adult worm burden (by 92.86%, and 95.24%, respectively), compared to the non-treated group. Both PZQ-based treatment regimens induced significant reduction in the males count (by 96.30%; the same reduction rate). Dapagliflozin monotherapy caused non-significant decrease in the males, females, and total worm counts (by 11.11%, 20%, and 14.29%, respectively), compared to infected untreated mice (Table 2).

#### Hepatic egg load

Treatment of *S. mansoni*-infected mice with PZQ alone or combined with DAPA significantly decreased the hepatic egg load (by 64.59%, and 66.43%, respectively). Moreover, DAPA dosing regimen caused significant reduction in the liver egg load (by 47.56%), compared to control mice (Table 2).

**Table 2:** Adult worm burden and hepatic egg load among different *Schistosoma mansoni*-infected mice groups, treated with praziquantel and dapagliflozin as mono- or combined therapy.

Mice groups	Adult worm burden			Hepatic egg load/gm × 10^3 b^
	Male ^a^	Female ^a^	Total ^a^	
Infected non-treated (n = 6)	13.5: 11–14	7.5: 6–10	21: 18–23	12.51 ± 0.64
DMSO (n = 5)	12: 10–14	7: 5–9	19: 17–23	12.13 ± 0.41
PZQ (n = 6)	0.5 (96.30) ^*^: 0–1	1.5 (80%) ^*^: 0–2	1.5 (92.86) ^*^: 0–3	4.43 ± 0.91 (64.59) ^**, ***^
DAPA (n = 5)	12 (11.11): 10–13	6 (20): 6–8	18 (14.29): 17–20	6.56 ± 2.52 (47.56) ^**^
DAPA + PZQ (n = 6)	0.5 (96.30) ^*^: 0–1	0.5 (93.33) ^*^: 0–1	1 (95.24) ^*^: 0–2	4.20 ± 1.12 (66.43) ^**, ***^
*P*-value	*P* ≤ 0.001	*P* ≤ 0.001	*P* ≤ 0.001	*P*≤ 0.001

^a^ Kruskal Wallis (KW) test. Data are expressed as medians: minimum-maximum

^b^ Analysis of variance (ANOVA) test. Data are expressed as means ± standard deviation

Values enclosed between parentheses represent the percentage of reduction, compared to infected non-treated group

DAPA: dapagliflozin; DMSO: dimethyl sulfoxide; PZQ: praziquantel

^*^ Significant difference against infected non-treated, DMSO-, and DAPA-treated groups at *P*˂0.01

^**^ Significant difference against infected non-treated, and DMSO-treated groups at *P*˂0.001

^***^ Significant difference against DAPA-treated group at *P*˂0.05

#### Oogram pattern

Administration o PZQ to *S. mansoni*-infected mice significantly reduced the percent of immature eggs (7.60), and increased the percent of dead eggs (49.48), in segments examined from the small intestine, compared to infected untreated group. Addition of PZQ to DAPA significantly increased the percent of mature and dead eggs (49.91 and 44.21, respectively), and significantly decreased the percent of immature eggs (5.88), compared to the control mice (Table 3).

**Table 3:** Oogram pattern among different *Schistosoma mansoni*-infected mice groups, treated with praziquantel and dapagliflozin as mono- or combined therapy.

Mice groups	Oogram pattern		
	Immature % ^a^	Mature % ^a^	Dead % ^a^
Infected non-treated (n = 6)	61.58 ± 11.17	38.42 ± 11.17	0 ± 0
DMSO (n = 5)	59.13 ± 5.15	38.07 ± 5.32	2.80 ± 1.27
PZQ (n = 6)	7.60 ± 4.45 ^*^	42.92 ± 4.25	49.48 ± 4.51 ^*^
DAPA (n= 5)	55.42 ± 9.81	34.33 ± 7.01	10.25 ± 4.33
DAPA + PZQ (n = 6)	5.88 ± 9.12 ^*^	49.91 ± 14.17 ^**^	44.21 ± 17.15 ^*^
*P*-value	*P* = 0.092	*P* ≤ 0.001	*P*≤ 0.001

^a^ Analysis of variance (ANOVA) test. Data are expressed as means ± standard deviation

DAPA: dapagliflozin; DMSO: dimethyl sulfoxide; PZQ: praziquantel

^*^ Significant difference against infected non-treated, DMSO-, and DAPA-treated groups at *P*˂ 0.001

^**^ Significant difference against infected non-treated, DMSO-, and DAPA-treated groups at *P*˂ 0.05

### Biochemical parameters

#### Assay of reduced glutathione and nitric oxide levels in liver homogenates

Significant increase in the levels of GSH and NO (by 60.73% and 61.11%, respectively) was demonstrated in the mice group given combined DAPA and PZQ therapy. In contrast, PZQ monotherapy caused non-significant increase in GSH and NO levels (by 4.73% and 11.90%, respectively), while DAPA regimen caused non-significant increase in GSH and NO levels (by 22.18% and 26.19%, respectively) (Table 4).

**Table 4:** Reduced glutathione and nitric oxide levels in liver homogenates among different *Schistosoma mansoni*-infected mice groups, treated with praziquantel and dapagliflozin as mono- or combined therapy.

Mice groups	Reduced glutathione (µmol/g) ^a^	Nitric oxide (µmol/g) ^a^
Infected non-treated (n = 6)	2.75 ± 0.91	2.52 ± 0.36
DMSO (n = 5)	2.72 ± 0.75	2.66 ± 0.27
PZQ (n = 6)	2.88 ± 0.26 (+ 4.73)	2.82 ± 0.30 (+ 11.90)
DAPA (n = 5)	3.36 ± 2.17 (+ 22.18)	3.18 ± 0.79 (+ 26.19)
DAPA + PZQ (n = 6)	4.42 ± 1.04 (+ 60.73) ^*^	4.06 ± 0.88 (+ 61.11) ^**^
*P*-value	*P*= 0.103	*P* ≤ 0.001

^a^ Analysis of variance (ANOVA) test. Data are expressed as means ± standard deviation

Values enclosed between parentheses represent the percentage of increase, compared to infected non-treated group; + increase

DAPA: dapagliflozin; DMSO: dimethyl sulfoxide; PZQ: praziquantel

^*^ Significant difference against infected non-treated, DMSO-, and DAPA-treated groups at *P*˂ 0.05

^**^ Significant difference against infected non-treated, DMSO-, PZQ-, and DAPA-treated groups *P*˂0.05

### Histopathological studies

#### Parenchymatous changes and portal tract inflammation and expansion

**Fig. 1:**
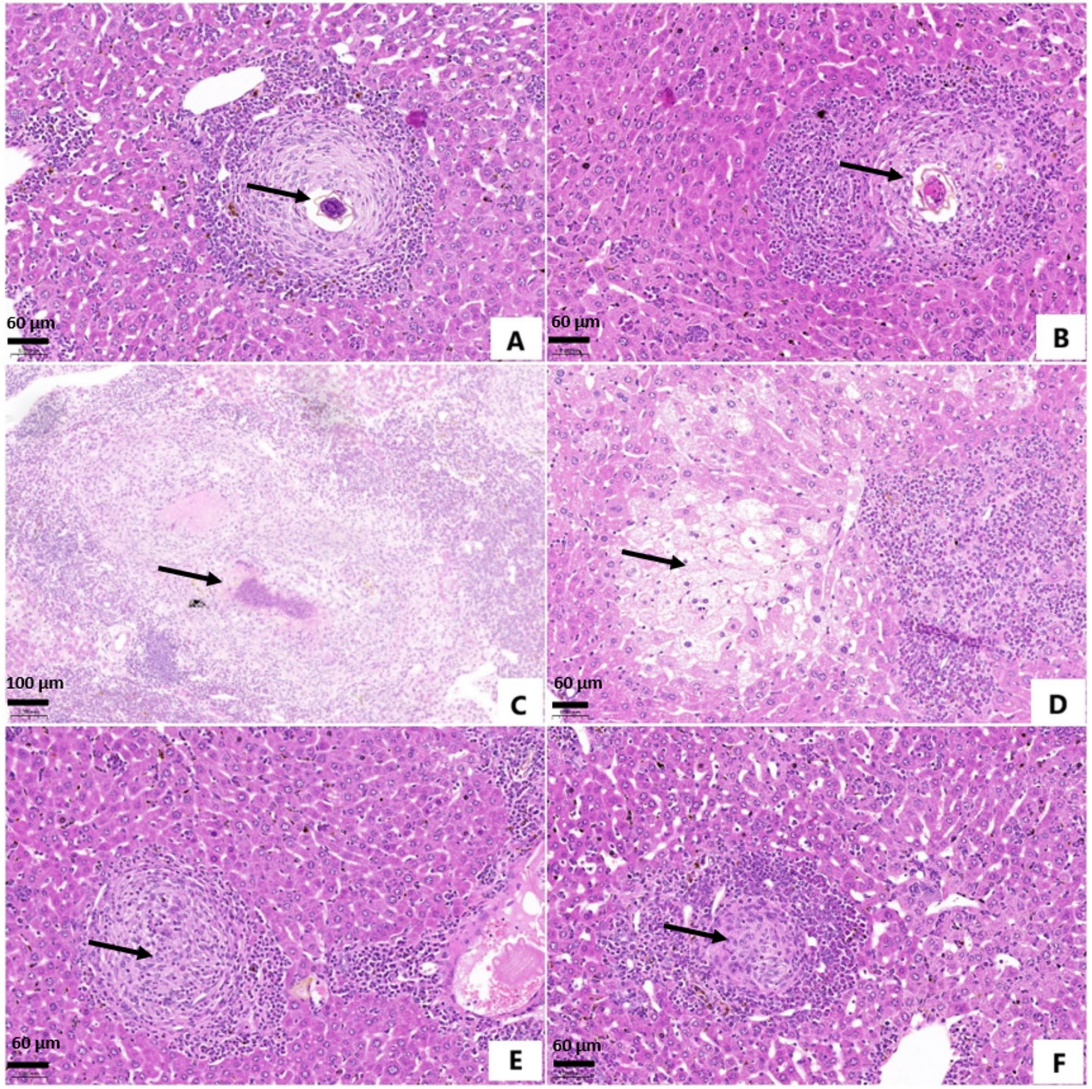
Photomicrographs of liver sections of different *Schistosoma mansoni*-infected mice groups, euthanized 10 weeks post infection. (Haematoxylin and Eosin; A, B, D, E, F: ×200, C: ×100). (**A**) Infected non-treated mice showing large irregularly outlined granuloma with massive inflammatory cellular infiltrate, encircling a living ovum (arrow).(**B**) Dimethyl sulfoxide-treated mice showing large irregularly outlined granuloma with massive inflammatory cellular infiltrate, encircling a living ovum (arrow).(**C**) Praziquantel-treated mice showing large irregularly outlined granuloma with massive inflammatory cellular infiltrate, encircling adead ovum (arrow).(**D**) Praziquantel-treated mice showing massive hydropic degeneration of hepatocytes (arrow) with massive inflammatory cellular infiltrate. (**E**) Dapagliflozin-treated mice showing small-sized circumscribed granuloma (arrow) with reduced inflammatory cellular infiltrate, encircling a partially degenerated ovum.(**F**) Mice treated with dapagliflozin and praziquantelshowing small-sized circumscribed granuloma (arrow) with reduced inflammatory cellular infiltrate, encircling a dead ovum.

Examination of liver sections from infected untreated mice showed marked lobular inflammation and focal necrosis, marked portal tract inflammation and expansion, and no hydropic degeneration. Mice treated with PZQ did not demonstrate significant amelioration of histopathological changes. In addition, sections showed hydropic degeneration (Fig. 1D), compared to non-treated mice. Histopathological examination of sections from mice administered DAPA dosing protocol and in combination with PZQ displayed significant alleviation. Besides, liver tissues showed no hydropic degeneration (Table 5).

#### Hepatic granulomas

**Fig. 2:**
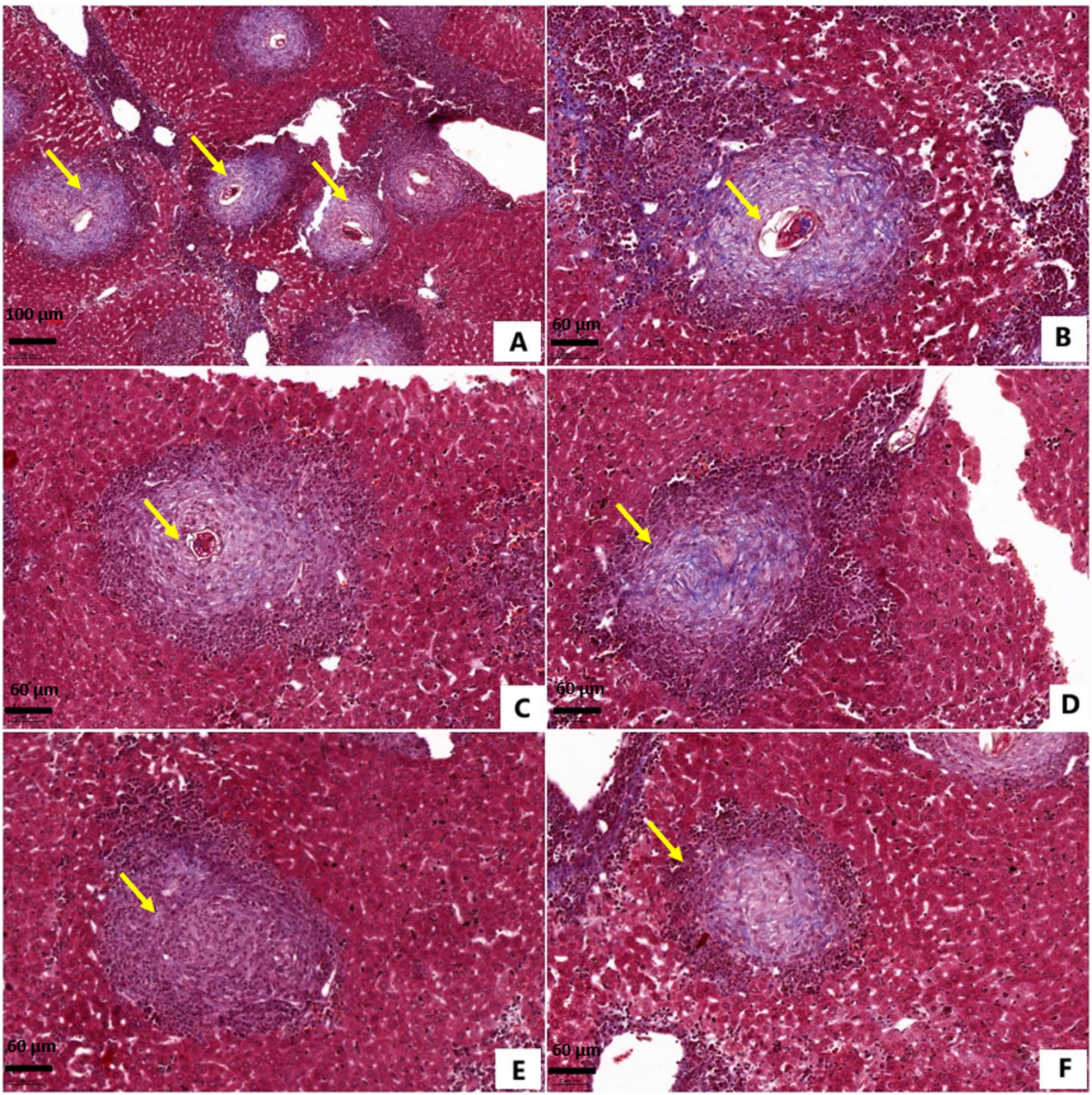
Photomicrographs of liver sections of different *Schistosoma mansoni*-infected mice groups, euthanized 10 weeks post infection.(Masson’s trichrome; A: ×100, B, C, D, E, F: ×200). (**A**,**B**) Infected non-treated mice showing large irregularly outlinedfibrocellular granulomas (arrows), encircling a living ovum.(**C**) Dimethyl sulfoxide-treated mice showing large irregularly-outlined fibrocellular granuloma, encircling a living ovum (arrow). (**D**) Praziquantel-treated mice showing large irregularly-outlined fibrocellular granuloma (arrow), encircling a dead ovum. (**E**) Dapagliflozin-treated mice showing small-sized fibrocellular granuloma(arrow), encircling a partially degenerated ovum. (**F**) Mice treated with dapagliflozin and praziquantel showing small-sized fibrocellular granuloma (arrow), encircling a dead ovum.

Liver sections of *S. mansoni*-infected untreated and DMSO-treated mice showed abundant large-sized irregularly outlined fibrocellular granulomas, encircling living ova (Figs. 1A and B and 2A and B, and 2C), with a mean count of 24.06 ± 3.89 and 23.13 ± 2.23, respectively, and a mean diameter of 522.22 ± 50.18 μm, and 506.67 ± 36.51 μm, respectively (Tables 5 and 6). Granulomas were surrounded with extensive inflammatory cellular infiltrate of eosinophils, macrophages, monocytes, fibroblasts, lymphocytes, and plasma cells.

Liver section from PZQ-treated mice showed moderate to marked inflammatory cellular infiltrate, and a reduced number of large-sized irregularly outlined fibrocellular granulomas, surrounding dead ova (Figs. 1C and 2D). Significant reduction in granuloma count by 48.05%, and non-significant reduction in granuloma diameter by 5.32%, compared to infected untreated mice, were demonstrated (Tables5 and 6).

Administration of DAPA alone and in combination with PZQ significantly attenuated the inflammatory cellular infiltrate. Sections showed small-sized fibrocellular granulomas, encircling partially degenerated or dead ova (Figs. 1E and F and 2E, and F), and significant reductions in the granuloma count by 38.20% and 57.27%, respectively, and diameter by 28.52%, and 27.66%, respectively, compared to the control group (Tables 5 and 6).

**Table 5:** Histopathological changes among different *Schistosoma mansoni*-infected mice groups, treated with praziquantel and dapagliflozin as mono- or combined therapy.

Histopathological changes	Score	Mice groups ^a^					*P*-value
		Infected non-treated (*n* = 6) ^***^	DMSO (*n* = 5) ^***^	PZQ (*n* = 6)	DAPA (*n* = 5) ^*, ***^	DAPA + PZQ (*n* = 6) ^*, **, ***^	
Lobular inflammation	None	–	–	–	–	–	P = 0.001
	Mild	–	–	–	–	–	
	Moderate			2 (33.33%)	4 (80%)	5 (83.33%)	
	Marked	6 (100%)	5 (100%)	4 (66.67%)	1 (20%)	1 (16.67%)	
Focal necrosis	None	–	–	–	–	–	
	Minimal	–	–	–	-	–	
	Mild	–	–	–	–	–	
	Moderate	–	–	2 (33.33%)	4 (80%)	5 (83.33%)	
	Marked	6 (100%)	5 (100%)	4 (66.67%)	1 (20%)	1 (16.67%)	
Portal tract inflammation and expansion	None	–	–	–	–	–	P ≤ 0.001
	Mild	–	–	1 (16.67%)	–	–	
	Moderate	–	–	1 (16.67%)	5 (100%)	5 (83.33%)	
	Marked	6 (100%)	5 (100%)	4 (66.66%)	–	1 (16.67%)	
Hydropic degeneration	None	6 (100%)	5 (100%)	–	5 (100%)	6 (100%)	P ≤ 0.001
	Minimal	–	–	1 (16.67%)	–	–	
	Mild	–	–	–	–	–	
	Moderate	–	–	2 (33.33%)	–	–	
	Marked	–	–	3 (50%)	–	–	
Inflammatory cellular infiltrate	Minimal	–	–	–	–	–	P = 0.003
	Mild	–	–	–	–	2 (33.33%)	
	Moderate	–	–	2 (33.33%)	4 (80%)	3 (50%)	
	Marked	6 (100%)	5 (100%)	4 (66.67%)	1 (20%)	1 (16.67%)	

^a^ Monte Carlo test. Data are expressed as number and percent

DAPA: dapagliflozin; DMSO: dimethyl sulfoxide; PZQ: praziquantel

^*^ Significant difference against infected non-treated, and DMSO-treated groups at *P*˂ 0.05 (lobular inflammation, focal necrosis, portal tract inflammation and expansion, inflammatory cellular infiltrate)

^**^ Significant difference against PZQ-treated group at *P*˂ 0.01 (inflammatory cellular infiltrate)

^***^ Significant difference against PZQ-treated group at *P*˂ 0.01 (hydropic degeneration)

**Table 6:** Hepatic granuloma count and diameter among different *Schistosoma mansoni*-infected mice groups, treated with praziquantel and dapagliflozin as mono- or combined therapy.

Mice groups	Granuloma count ^a^	Granuloma diameter (µm) ^a^
Infected non-treated (n = 6)	24.06 ± 3.89	522.22 ± 50.18
DMSO (n = 5)	23.13 ± 2.23	506.67 ± 36.51
PZQ (n = 6)	12.50 (48.05) ± 7.91^*^	494.44 ± 32.77 (5.32)
DAPA (n = 5)	14.87 (38.20) ± 5.43 ^*^	373.33 ± 36.51 (28.52) ^**^
DAPA + PZQ (n = 6)	10.28 (57.27) ± 2.96^*^	377.78 ± 17.21 (27.66) ^**^
*P*-value	*P* ≤ 0.001	*P* ≤ 0.001

^a^ Analysis of variance (ANOVA) test. Data are expressed as means ± standard deviation

Values enclosed between parentheses represent the percentage of reduction, compared to infected non-treated group

DAPA: dapagliflozin; DMSO: dimethyl sulfoxide; PZQ: praziquantel

^*^ Significant difference against infected non-treated, and DMSO-treated groups at *P*˂ 0.05

^**^ Significant difference against infected non-treated, DMSO-, and PZQ-treated groups at *P*˂ 0.001

### Immunohistochemical study of cleaved-caspase−3

**Fig. 3:**
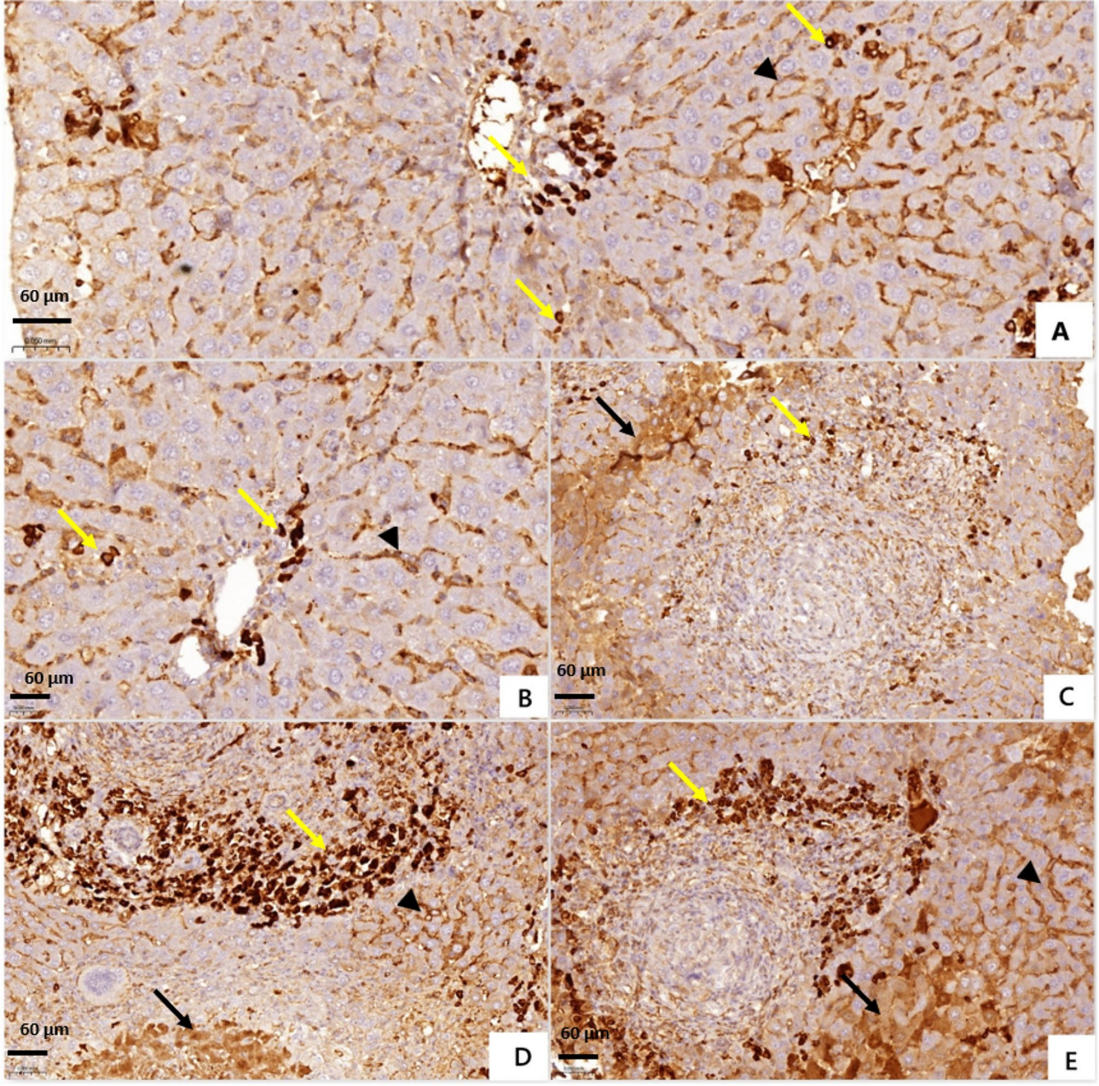
Photomicrographs of liver sections of different *Schistosoma mansoni*-infected mice groups, euthanized 10 weeks post infection. (Immunohistochemical staining for cleaved-caspase-3, Diaminobenzidine ×200). (**A**) Infected non-treated mice showing negative expression in hepatocytes, positive expression in Kupffer cells (arrow’s head), and weak expression in inflammatory cells in portal areas and in-between hepatocytes (yellow arrows).(**B**) Dimethyl sulfoxide-treated mice showing negative expression in hepatocytes, positive expression in Kupffer cells (arrow’s head) and weak expression in inflammatory cells in portal areas and in-between hepatocytes (yellow arrows).(**C**) Praziquantel-treated mice showing weak expression in hepatocytes(black arrow), and weak expression in inflammatory cells(yellow arrow).(**D**) Dapagliflozin-treated mice showing weakexpression in hepatocytes(black arrow), positive expression in Kupffer cells (arrow’s head), and strong expression in inflammatory cells in portal areas and in-between hepatocytes(yellow arrow).(**E**) Mice treated with dapagliflozinand praziquantelshowing weak expression in hepatocytes(black arrow), positive expression in Kupffer cells (arrow’s head), and strong expression in inflammatory cells(yellow arrow).

Examination of liver sections from *S. mansoni*-infected non-treated and DMSO-treated mice for cleaved-caspase-3 antibody revealed negative expression in hepatocytes (trace), and positive expression in 10–20% of inflammatory cells in portal areas and in-between hepatocytes (weak 1+). Sections from PZQ-treated mice showed positive expression in 15–20% of hepatocytes (weak1+), and positive expression in 5–10% of inflammatory cells (weak 1+). Administration of DAPA as monotherapy and in combination with PZQ was associated with positive expression in 15–20% of hepatocytes (weak 1+), and positive expression in > 75% of inflammatory cells (strong 3+) (Table 7; Fig. 3). Different localization of cleaved-caspase-3 was observed; immunoreactivity was confined to the cytoplasm in hepatocytes, whereas in inflammatory cells, staining was detected in the cytoplasm and nuclei.

**Table 7:** Immunohistochemistry score of cleaved-caspase-3 expression among different *Schistosoma mansoni*-infected mice groups, treated with praziquantel and dapagliflozin as mono- or combined therapy.

Cells	Score	Mice groups ^a^					*P*-value
		Infected non-treated (*n* = 6)	DMSO (*n* = 5)	PZQ (*n* = 6) ^*^	DAPA (*n* = 5) ^*, **^	DAPA + PZQ (*n* = 6) ^*, **^	
Hepatocytes	Trace	6 (100%)	5 (100%)	–	–	–	*P* ≤ 0.001
	Weak (1+)	–	–	6 (100%)	5 (100%)	6 (100%)	
	Moderate (2+)	–	–	–	–	–	
	Strong (3+)	–	–	–	–	–	
Inflammatory cells	Trace	–	–	–	–	–	
	Weak (1+)	6 (100%)	5 (100%)	6 (100%)	–	–	
	Moderate (2+)	–	–	–	–	–	
	Strong (3+)	–	–	–	5 (100%)	6 (100%)	

^a^ Monte Carlo test. Data are expressed as number and percent

DAPA: dapagliflozin; DMSO: dimethyl sulfoxide; PZQ: praziquantel

^*^ Significant difference against infected non-treated, and DMSO-treated groups at *P*˂ 0.01 (hepatocytes)

^**^ Significant difference against infected non-treated, DMSO-treated, and PZQ-treated groups at *P*˂ 0.01(inflammatory cells)

## Discussion

Since schistosomicidal drugs lack efficacy in reversing established liver pathology^36^, the development of new therapeutic strategies targeting key regulators of hepatic fibrosis in schistosomiasis remains a priority.

To the best of our knowledge, this is the first study investigating the effects of the oral anti-diabetic drug DAPA on *S. mansoni*-induced liver pathology. This study was conducted to scrutinize its anti-oxidant, anti-inflammatory, and apoptosis-regulatory effects using *S. mansoni*-infected BALB/c female mice.

In the present study, administration of PZQ alone or in combination with DAPA significantly decreased adult worm burden, and hepatic egg load, compared to the control mice, indicating that DAPA did not hinder the schistosomicidal efficacy of PZQ. The significant reduction in hepatic egg load in PZQ-treated mice, compared to infected untreated mice, is caused by eradication of adult *S. mansoni* parasites, and cessation of egg deposition in response to PZQ therapy^28^. The reduction in hepatic egg load in response to DAPA monotherapy, in spite of the non-significant reduction in adult worm burden, can be explained by the immunomodulatory effect of DAPA^20,22^. This effect is linked to tissue remodeling and structural changes, and may partially explain the reduction in hepatic egg load, by enhancing egg expulsion or excretion.

Concerning oogram pattern, the significant increase in the percent of dead eggs, and the significant reduction in the percent of immature eggs, in *S. mansoni*-infected mice that received PZQ in combination with DAPA, implies that DAPA did not impair PZQ anti-schistosomal activity. In our hands, DAPA monotherapy did not significantly alter the oogram pattern, compared with infected non-treated mice. Additionally, it did not significantly decrease adult worm count. This signifies that the drug alone has no anti-schistosomal activity.

Despite its schistosomicidal efficacy, PZQ monotherapy did not increase GSH or NO levels, as it did not significantly ameliorate histopathological changes. The use of DAPA in combination with PZQ, induced significant increase in the levels of GSH and NO. This can be explained by the significant reduction in hepatic egg load, as well as the significant alleviation of histopathological changes, in response to the combined regimen.

The impact of DAPA on mitigating oxidative stress was investigated by Hassan et al.^20^, who reported that DAPA treatment caused significant reduction in hepatic malondialdehyde, and significant increase in hepatic catalase and superoxide dismutase levels, compared to the control rats. Also, Tang et al.^21^ documented that DAPA administration decreased hepatic production of superoxide anion radicals, and renal NAPDH oxidase generation and activation. Besides, DAPA was proved to restore the reduced NO bioavailability in tumor necrosis factor-α (TNF-α)-stimulated human coronary arterial endothelial cells, through inhibiting ROS production^37^. Furthermore, administration of DAPA was shown to decrease ROS production, increase superoxide dismutase activity and ATP levels, and reduce malondialdehyde levels in a rat model of coronary microembolization^38^.

Treatment of *S. mansoni*-infected mice with PZQ dosing regimen did not significantly ameliorate histopathological changes, compared to the untreated mice group. Liver sections also showed hydropic degeneration; implying a drug hepatotoxic effect^39^. The alleviation of liver pathology in DAPA-treated groups, compared to infected untreated mice can be attributed to the anti-inflammatory effect of DAPA. Moreover, the absence of hydropic degeneration in DAPA-treated mice denotes that the drug has no hepatotoxic effect, and could also attenuate the hepatotoxic effect of PZQ, indicating a potential hepatoprotective efficacy. The amelioration of histopathological changes in response to DAPA therapy was proved by Hassan et al.^20^ and Han et al.^40^, in rat models of common bile duct ligation and sepsis-induced heart injury, respectively.

In the current study, the significant reduction in granuloma count, in mice groups treated with PZQ alone and in combination with DAPA, compared to infected untreated mice, is related to PZQ effect on adult worms and hepatic egg load. On the other hand, the significant reduction in granuloma count following DAPA therapy, compared to control mice, can be possibly explained by delayed granuloma formation as a result of reduced TNF levels^41^, induced by DAPA administration^42^.

Regarding granuloma diameter, the non-significant reduction after PZQ therapy can be explained by PZQ failure to reduce inflammatory cellular infiltrate. In contrast, DAPA dosing regimens significantly decreased the inflammatory cellular infiltrate. The anti-inflammatory activity of DAPA was documented to be caused by inhibition of interleukin-1β, interleukin-18, and TNF-α^42^. In addition, DAPA decreases nuclear factor-kappa B (NF-κB) signalling and interferon-γ^43,44^, with subsequent reduction in granuloma diameter^45^. Our findings are lower than those obtained by Salama et al.^46^ who assessed the effects of metformin on *S. mansoni*-infected mice. This can be ascribed to difference in mice strain and sex, as well as difference in infective dose of cercariae, drugs nature, and dosing regimens.

In schistosomiasis, peri-portal collagen deposition and obstruction of blood flow causes portal hypertension, hepatomegaly, and splenomegaly^47^. In the present study, *S. mansoni*-infected mice given PZQ alone and in combination with DAPA showed significant reduction in liver and spleen indices. This reduction can be explained by the significantly decreased granuloma count. Additionally, the significant reduction in liver and spleen indices following DAPA monotherapy can be attributed to its anti-inflammatory effect which subsequently amended hepatic perfusion, and the significantly reduced liver egg load and portal tract inflammation and expansion, as well as the significantly reduced granuloma count^48^. Similarly, Shaaban et al.^22^ archived reduced liver index following DAPA administration in combination with metformin or pioglitazone in a rat model of non-alcoholic fatty liver disease.

In our experiment, the absence of caspase-3 expression in hepatocytes in untreated mice can be explained by the fact that schistosomal granulomas are necrotic^49^. Weak caspase-3 expression in inflammatory cells can be attributed to the effects of certain molecules, such as soluble egg antigen which can induce apoptosis in T lymphocytes^50^.

Herein, the induction of apoptosis in damaged hepatocytes and inflammatory cells in DAPA-treated mice may be caused by a possible senolytic effect^51,52^ of DAPA on these senescent cells; since inflammatory cytokines induce cellular senescence^53^. Meanwhile, the alleviation of oxidative stress induced by DAPA therapy (as evidenced by the increased levels of GSH and NO) protected healthy hepatocytes against damage^54^. Therefore, DAPA hepatoprotective efficacy in schistosomiasis mansoni-induced hepatopathy is partially related to regulation of apoptosis, and is caspase-3 dependent. It is important to note that induction of caspase-3 can clarify the diminished inflammatory response, in DAPA-treated groups, as it causes cleavage of pro-interleukin-1β^55^.

Mice treated with PZQ dosing regimen showed weak caspase-3 expression in inflammatory cells. This may reflect an imperfect fine tuning of the apoptotic response in the liver cells, and can partially explain the incomplete hepatoprotective effect of PZQ, compared to DAPA-treated groups.

It is known that persistent inflammation is associated with the aggregation of dendritic cells, macrophages, and monocytes, and the release of inflammatory mediators, such as interleukin-1, interleukin-6, and TNF-α, which further exacerbate the inflammatory response^56^. Moreover, damage-associated molecular patterns generated in response to hepatocyte injury provoke a TH2-polarized immune response. This, in turn, endorses the proliferation and activation of HSCs, and promotes liver fibrosis^57^. Therefore, reducing the release of inflammatory mediators through therapeutic interventions is coupled with attenuated inflammation and fibrogenesis signaling pathways^58^.

No preceding studies investigated the effect of DAPA on caspase-3 expression in a murine model of schistosomiasis to compare our findings against. Interestingly, a number of research studies have shown protective effects following caspase-3 induction or attenuation, in response to the same agent; DAPA. High levels of caspase-3 and increased apoptosis were demonstrated by El-Demerdash et al.^59^, Elkhyat et al.^60^, and Jang et al.^61^, in a rat model of arthritis, Ehrlich ascites carcinoma cell lines, and Caki-1 human renal carcinoma cells, respectively.

Contrastively, attenuation of apoptosis and reduced caspase-3 in response to DAPA therapy was recorded in cardiomyocytes in coronary microembolization^38^ and sepsis-induced cardiomyopathy^40^, and in the abdominal macrophages of diabetic mice^62^. This discrepancy may be explained by differences in disease models, and affected organs or cell lines, as well as interaction with different molecules and signalling pathways.

It is worth citing that appropriate induction of apoptosis in HSCs can impede ECM deposition, and is considered a promising approach against liver fibrosis^4^. In the liver, NF-κB promotes HSCs survival through transcriptional upregulation of transformation growth factor-β and TNF-α; which function as anti-apoptotic proteins^63,64^. Therefore, the effect of DAPA on HSCs, mediated through its inhibitory effect on NF-κB signaling^43^ merits further study.

## Conclusions

This study highlights, for the first time, the potential beneficial effects of the anti-diabetic drug DAPA in *S. mansoni*-infected mice. The hepatoprotective effects of DAPA could be credited to its efficacy in targeting inflammation, oxidative stress, and apoptosis axes. Our findings underlined the use of DAPA as a convenient adjuvant agent to PZQ to alleviate schistosomal hepatopathy. Additional studies are recommended to unveil other mechanisms responsible for the hepatoprotective effects of DAPA on schistosomiasis-induced liver fibrosis, as well as on other models of fibroproliferative disorders. Monitoring of blood glucose levels, cytokine profiling, and in vitro studies should be incorporated into future research, for better elucidation of the metabolic, hepatoprotective, and immunomodulatory effects of DAPA.

### Limitations of the study

Owing to logistical and financial constraints, other control groups (uninfected mice, the uninfected DAPA-treated, and infected Cremophor El 2%-treated) were not included in the current research. Comparison was primarily focused on treated *versus* non-treated mice.

## Data Availability

The datasets used and/or analyzed during the current work are available upon reasonable request from the corresponding author.

## References

[1] Li, Q. al. Global trends of schistosomiasis burden from 1990 to 2021 across 204 countries and territories: Findings from GBD 2021 study. *Acta Trop.* **261**, 107504 (2025). 39675411 10.1016/j.actatropica.2024.107504

[2] Acharya, P., Chouhan, K., Weiskirchen, S. & Weiskirchen, R. Cellular mechanisms of liver fibrosis. *Front. Pharmacol.* **12**, 671640 (2021). 34025430 10.3389/fphar.2021.671640 PMC8134740

[3] Liu, Z., Zhang, L., Liang, Y. & Lu, L. Pathology and molecular mechanisms of *Schistosoma japonicum*-associated liver fibrosis. *Front. Cell. Infect. Microbiol.* **12**, 1035765 (2022). 36389166 10.3389/fcimb.2022.1035765 PMC9650140

[4] Zhang, C. Y., Yuan, W. G., He, P., Lei, J. H. & Wang, C. X. Liver fibrosis and hepatic stellate cells: Etiology, pathological hallmarks and therapeutic targets. *World J. Gastroenterol.* **22** (48), 10512–10522 (2016). 28082803 10.3748/wjg.v22.i48.10512 PMC5192262

[5] Mittal, M., Siddiqui, M. R., Tran, K., Reddy, S. P. & Malik, A. B. Reactive oxygen species in inflammation and tissue injury. *Antioxid. Redox Signal.* **20** (7), 1126–1167 (2014). 23991888 10.1089/ars.2012.5149 PMC3929010

[6] Pizzino, G. et al. Oxidative stress: Harms and benefits for human health. *Oxid. Med. Cell. Longev.* 8416763 (2017). (2017). 10.1155/2017/8416763 PMC5551541 28819546

[7] Forman, H. J. & Zhang, H. Targeting oxidative stress in disease: Promise and limitations of antioxidant therapy. *Nat. Rev. Drug Discov*. **20** (9), 689–709 (2021). 34194012 10.1038/s41573-021-00233-1 PMC8243062

[8] Rudrapal, M. et al. Dietary polyphenols and their role in oxidative stress-induced human diseases: Insights into protective effects, antioxidant potentials and mechanism(s) of action. *Front. Pharmacol.* **13**, 806470 (2022). 35237163 10.3389/fphar.2022.806470 PMC8882865

[9] Zhang, D., Zhang, Y. & Sun, B. The molecular mechanisms of liver fibrosis and its potential therapy in application. *Int. J. Mol. Sci.* **23**, 12572 (2022). 36293428 10.3390/ijms232012572 PMC9604031

[10] Romo, M. R. Cell death as part of innate immunity: Cause or consequence? *Immunology* **163** (4), 399–415 (2021). 33682112 10.1111/imm.13325 PMC8274179

[11] Lamkanfi, M., Declercq, W., Kalai, M., Saelens, X. & Vandenabeele, P. Alice in caspase land. A phylogenetic analysis of caspases from worm to man. *Cell. Death Differ.* **9** (4), 358–361 (2002). 11965488 10.1038/sj.cdd.4400989

[12] Elmore, S. & Apoptosis A review of programmed cell death. *Toxicol. Pathol.* **35** (4), 495–516 (2007). 17562483 10.1080/01926230701320337 PMC2117903

[13] Wu, Y., Zhao, D., Zhuang, J., Zhang, F. & Chun Xu, C. Caspase–8 and caspase–9 functioned differently at different stages of the cyclic stretch-induced apoptosis in human periodontal ligament cells. *PLoS One*. **11** (12), e0168268 (2016). 27942018 10.1371/journal.pone.0168268 PMC5152893

[14] Nadendla, E. K., Tweedell, R. E., Kasof, G., Kanneganti, T. D. & Caspases Structural and molecular mechanisms and functions in cell death, innate immunity, and disease. *Cell. Discov*. **11** (1), 42 (2025). 40325022 10.1038/s41421-025-00791-3 PMC12052993

[15] Tentolouris, A., Vlachakis, P., Tzeravini, E., Eleftheriadou, I. & Tentolouris, N. SGLT2 inhibitors: A review of their antidiabetic and cardioprotective effects. *Int. J. Environ. Res. Public. Health*. **16** (16), 2965 (2019). 31426529 10.3390/ijerph16162965 PMC6720282

[16] Perry, R. J. & Shulman, G. I. Sodium-glucose cotransporter–2 inhibitors: Understanding the mechanisms for therapeutic promise and persisting risks. *J. Biol. Chem.* **295** (42), 14379–14390 (2020). 32796035 10.1074/jbc.REV120.008387 PMC7573269

[17] Zelniker, T. A. & Braunwald, E. Mechanisms of cardiorenal effects of sodium-glucose cotransporter 2 inhibitors: JACC state-of-the-art review. *J. Am. Coll. Cardiol.* **75** (4), 422–434 (2020). 32000955 10.1016/j.jacc.2019.11.031

[18] Maccari, R. & Ottanà, R. Sodium-glucose cotransporter inhibitors as antidiabetic drugs: Current development and future perspectives. *J. Med. Chem.* **65** (16), 10848–10881 (2022). 35924548 10.1021/acs.jmedchem.2c00867 PMC9937539

[19] Hu, J., Teng, J., Hui, S. & Liang, L. SGLT–2 inhibitors as novel treatments of multiple organ fibrosis. *Heliyon* **10** (8), e29486 (2024). 10.1016/j.heliyon.2024.e29486 PMC11031788 38644817

[20] Hassan, H. A. et al. Dapagliflozin dampens liver fibrosis induced by common bile duct ligation in rats associated with the augmentation of the hepatic Sirt1/AMPK/PGC1α/FoxO1 axis. *Toxicol. Appl. Pharmacol.* **489**, 116991 (2024). 38871090 10.1016/j.taap.2024.116991

[21] Tang, L. et al. Dapagliflozin slows the progression of the renal and liver fibrosis associated with type 2 diabetes. *Am. J. Physiol. Endocrinol. Metab.* **313** (5), E563–E576 (2017). 28811292 10.1152/ajpendo.00086.2017

[22] Shaaban, H. H. et al. Metformin, pioglitazone, dapagliflozin and their combinations ameliorate manifestations associated with NAFLD in rats *via* anti-inflammatory, anti-fibrotic, anti-oxidant and anti-apoptotic mechanisms. *Life Sci.* **308**, 120956 (2022). 36103959 10.1016/j.lfs.2022.120956

[23] Nair, A. B. & & Jacob, S. A simple practice guide for dose conversion between animals and human. *J. Basic. Clin. Pharm.* **7** (2), 27–31 (2016). 27057123 10.4103/0976-0105.177703 PMC4804402

[24] Gönnert, R. & Andrews, P. Praziquantel, a new broad-spectrum antischistosomal agent. *Z. Parasitenkund*. **52**, 129–150 (1977). 10.1007/BF00389899 410178

[25] Smithers, S. R. & Terry, R. J. The infection of laboratory hosts with cercariae of *Schistosoma mansoni* and the recovery of the adult worms. *Parasitology* **55** (4), 695–700 (1965). 4957633 10.1017/s0031182000086248

[26] Arifin, W. N. & Zahiruddin, W. M. Sample size calculation in animal studies using resource equation approach. *Malays J. Med. Sci.* **24** (5), 101–105 (2017). 29386977 10.21315/mjms2017.24.5.11 PMC5772820

[27] Cheever, A. W. A quantitative post-mortem study of schistosomiasis mansoni in man. *Am. J. Trop. Med. Hyg.* **17** (1), 38–64 (1968). 5637021 10.4269/ajtmh.1968.17.38

[28] Pellegrino, J., Oliveira, C. A., Faria, J. & Cunha, A. S. New approach to the screening of drugs in experimental schistosomiasis *mansoni* in mice. *Am. J. Trop. Med. Hyg.* **11**, 201–215 (1962). 14484966 10.4269/ajtmh.1962.11.201

[29] Tandra, S. et al. Presence and significance of microvesicular steatosis in nonalcoholic fatty liver disease. *J. Hepatol.* **55** (3), 654–659 (2011). 21172393 10.1016/j.jhep.2010.11.021 PMC3139780

[30] Suzuki, S. & Toledo-Pereyra, L. H. Monoclonal antibody to intercellular adhesion molecule 1 as an effective protection for liver ischemia and reperfusion injury. *Transplant. Proc.* 25 (6), 3325–3327 (1993). 7903499

[31] Knodell, R. G. et al. Formulation and application of a numerical scoring system for assessing histological activity in asymptomatic chronic active hepatitis. *Hepatology* **1** (5), 431–435 (1981). 7308988 10.1002/hep.1840010511

[32] Poo, J. L. et al. Semiquantitative histologic evaluation of the liver in patients after liver transplantation. *Transplant. Proc.* 24 (5), 1973–1975 (1992). 1412935

[33] von Lichtenberg, F. Host response to eggs of *S. mansoni*. I. Granuloma formation in the unsensitized laboratory mouse. *Am. J. Pathol.* **41** (6), 711–731 (1962). 13930476 PMC1949631

[34] Suvarna, K. S., Layton, C. & Bancroft, J. D. *Bancroft’s Theory and Practice of Histological Techniques* 8th edn (Elsevier, 2018).

[35] Huang, K. H. et al. Caspase–3, a key apoptotic protein, as a prognostic marker in gastric cancer after curative surgery. *Int. J. Surg.* **52**, 258–263 (2018). 29501797 10.1016/j.ijsu.2018.02.055

[36] Nono, J. K. et al. Investigating the antifibrotic effect of the antiparasitic drug praziquantel in *in vitro* and *in vivo* preclinical models. *Sci. Rep.* **10** (1), 10638 (2020). 32606340 10.1038/s41598-020-67514-4 PMC7327036

[37] Uthman, L. et al. Empagliflozin and dapagliflozin reduce ROS generation and restore NO bioavailability in tumor necrosis factor α-stimulated human coronary arterial endothelial cells. *Cell. Physiol. Biochem.* **53** (5), 865–886 (2019). 31724838 10.33594/000000178

[38] Li, T., Luo, C. J., Yi, Z. Q. & Li, L. Dapagliflozin attenuates myocardial inflammation and apoptosis after coronary microembolization in rats by regulating the SIRT1/NF-κB signaling pathway. *Front. Biosci. (Landmark Ed)*. **30** (3), 27082 (2025). 40152380 10.31083/FBL27082

[39] Shatat, A. R., Ahmed, O. B. & Ali, G. A. M. Liver protection from acetaminophen hepatotoxicity using copper(I)-nicotinic acid complex. *Egypt. J. Chem.* **65** (6), 111–120 (2022).

[40] Han, X. et al. Dapagliflozin ameliorates sepsis-induced heart injury by inhibiting cardiomyocyte apoptosis and electrical remodeling through the PI3K/Akt pathway. *Eur. J. Pharmacol.* **955**, 175930 (2023). 37479014 10.1016/j.ejphar.2023.175930

[41] Davies, S. J. et al. Involvement of TNF in limiting liver pathology and promoting parasite survival during schistosome infection. *Int. J. Parasitol.* **34** (1), 27–36 (2004). 14711587 10.1016/j.ijpara.2003.10.010 PMC2859728

[42] ElMahdy, M., Kh., Helal, M. G. & Ebrahim, T. M. Potential anti-inflammatory effect of dapagliflozin in HCHF diet- induced fatty liver degeneration through inhibition of TNF-α, IL–1β, and IL–18 in rat liver. *Int. Immunopharmacol.* **86**, 106730 (2020). 32599319 10.1016/j.intimp.2020.106730

[43] Quagliariello, V. et al. Sodium-glucose cotransporter 2 inhibitor dapagliflozin prevents ejection fraction reduction, reduces myocardial and renal NF-κB expression and systemic pro-inflammatory biomarkers in models of short-term doxorubicin cardiotoxicity. *Front. Cardiovasc. Med.* **11**, 1289663 (2024). 38818214 10.3389/fcvm.2024.1289663 PMC11138344

[44] Cai, A. et al. Dapagliflozin alleviates renal inflammation and protects against diabetic kidney diseases, both dependent and independent of blood glucose levels. *Front. Immunol.* **14**, 1205834 (2023). 38022502 10.3389/fimmu.2023.1205834 PMC10665888

[45] Rezende, S. A. et al. Mice lacking the gamma interferon receptor have an impaired granulomatous reaction to *Schistosoma mansoni* infection. *Infect. Immunol.* **65** (8), 3457–3461 (1997). 9234812 10.1128/iai.65.8.3457-3461.1997 PMC175489

[46] Salama, W. M., El-Naggar, S. A., Harras, S. F. & El-Said, K. An adjuvant effect of metformin as an anti-fibrotic agent when administered with the anti-schistosomal praziquantel in *Schistosoma mansoni* infected mice. *Trop. Biomed.* **38** (2), 205–213 (2021). 34172712 10.47665/tb.38.2.059

[47] Asta, A., Dahl, J., Bryant, D. & Ammar, H. An ancient disease hepatosplenic schistosomiasis. *J. Community Hosp. Intern. Med. Perspect.* **14** (2), 117–119 (2024). 38966512 10.55729/2000-9666.1327 PMC11221448

[48] Berhe, N., Myrvang, B. & Gundersen, S. G. Reversibility of schistosomal periportal thickening/fibrosis after praziquantel therapy: A twenty-six month follow-up study in Ethiopia. *Am. J. Trop. Med. Hyg.* **78** (2), 228–234 (2008). 18256420

[49] Choi, J. H. Histological and molecular evaluation of liver biopsies: A practical and updated review. *Int. J. Mol. Sci.* **26** (16), 7729 (2025). 40869049 10.3390/ijms26167729 PMC12387026

[50] Wang, Y. et al. Eggs of *Schistosoma japonicum* deposited in the spleen induce apoptosis of splenic T cells in C57BL/6 mice. *Parasitol. Res.* **124**, 31 (2025). 40059230 10.1007/s00436-025-08474-4 PMC11891099

[51] Katsuumi, G. et al. SGLT2 inhibition eliminates senescent cells and alleviates pathological aging. *Nat. Aging*. **4** (7), 926–938 (2024). 38816549 10.1038/s43587-024-00642-y PMC11257941

[52] Yesilyurt-Dirican, Z. E. et al. SGLT2 inhibitors as a novel senotherapeutic approach. *NPJ Aging*. **11** (1), 35 (2025). 40348751 10.1038/s41514-025-00227-y PMC12065912

[53] Ren, J. L., Pan, J. S., Lu, Y. P., Sun, P. & Han, J. Inflammatory signaling and cellular senescence. *Cell. Signal.* **21** (3), 378–383 (2009). 18992324 10.1016/j.cellsig.2008.10.011 PMC2870712

[54] Singh, R. & & Czaja, M. J. Regulation of hepatocyte apoptosis by oxidative stress. *J. Gastroenterol. Hepatol.* **22** (Suppl 1), S45–S48 (2007). 17567464 10.1111/j.1440-1746.2006.04646.x

[55] Kim, S. S. & & Seo, S. R. Caspase–3 targets pro-interleukin–1β (IL–1β) to restrict inflammation. *FEBS Lett.* **598** (11), 1366–1374 (2024). 38553939 10.1002/1873-3468.14864

[56] Lu, J. L., Yu, C. X. & Song, L. J. Programmed cell death in hepatic fibrosis: Current and perspectives. *Cell. Death Discov*. **9** (1), 449 (2023). 38086792 10.1038/s41420-023-01749-8 PMC10716404

[57] Vicentino, A. R. R. et al. Emerging role of HMGB1 in the pathogenesis of schistosomiasis liver fibrosis. *Front. Immunol.* **9**, 1979 (2018). 30258438 10.3389/fimmu.2018.01979 PMC6143665

[58] Hammerich, L. & Tacke, F. Hepatic inflammatory responses in liver fibrosis. *Nat. Rev. Gastroenterol. Hepatol.* **20** (10), 633–646 (2023). 37400694 10.1038/s41575-023-00807-x

[59] El-Demerdash, A. A., Darwish, S. F. & El-Derany, M. O. El-Demerdash, E. Dapagliflozin targets the crosstalk between apoptosis, autophagy, and Hedgehog signaling pathways through AMPK activation in the adjuvant-induced arthritic rat model. *Inflammopharmacology* **33** (6), 3157–3176 (2025). 40350466 10.1007/s10787-025-01750-w PMC12213983

[60] Elkhyat, S. M. M., Elmahdy, N. A., Elhusseiny, M. E. & Zidan, A. A. Impact of dapagliflozin on apoptosis and effector cytotoxic cells using breast cancer cells in mice. *Bull. Pharm. Sci. Assiut Univ.* **47** (1), 655–667 (2024).

[61] Jang, J. H., Lee, T. J., Sung, E. G., Song, I. H. & Kim, J. Y. Dapagliflozin induces apoptosis by downregulating cFILP_L_ and increasing cFILP_S_ instability in Caki–1 cells. *Oncol. Lett.* **24** (5), 401 (2022). 36276495 10.3892/ol.2022.13521 PMC9533662

[62] Xiong, S. X. et al. Dapagliflozin exerts anti-apoptotic effects by mitigating macrophage polarization *via* modulation of the phosphoinositide 3-kinase/protein kinase B signaling pathway. *World J. Diabetes*. **16** (2), 97287 (2025). 39959262 10.4239/wjd.v16.i2.97287 PMC11718488

[63] Saile, B., Matthes, N., Armouche, E., Neubauer, H., Ramadori, G. & K. & The bcl, NF-kappaB and p53/p21WAF1 systems are involved in spontaneous apoptosis and in the anti-apoptotic effect of TGF-beta or TNF-alpha on activated hepatic stellate cells. *Eur. J. Cell. Biol.* **80** (8), 554–561 (2001). 11561906 10.1078/0171-9335-00182

[64] Oakley, F. et al. Hepatocytes express nerve growth factor during liver injury: Evidence for paracrine regulation of hepatic stellate cell apoptosis. *Am. J. Pathol.* **163** (5), 1849–1858 (2003). 14578185 10.1016/S0002-9440(10)63544-4 PMC1892444

